# Proportional representation and incidence rate of repeat visits in ethnic minorities compared to native Dutch people under the age of 25 years in the Netherlands

**DOI:** 10.1016/j.jmh.2025.100344

**Published:** 2025-07-26

**Authors:** Y.J. Evers, A. Verhaegh, A. Ibrahim, C. Peters, N.H.T.M. Dukers-Muijrers, R. Reijs, C.J.P.A. Hoebe

**Affiliations:** a Department of Social Medicine, Care and Public Health Research Institute (CAPHRI), Maastricht University/Maastricht UMC+, Maastricht, the Netherlands; b Department of Sexual Health, Infectious Diseases and Environmental Health, Living Lab Public Health Mosa, South Limburg Public Health Service, Heerlen, the Netherlands; c Department of Sexual Health, Municipal Public Health Service Rotterdam-Rijnmond, Rotterdam, the Netherlands; d Department of Health Promotion, Care and Public Health Research Institute (CAPHRI), Maastricht University/Maastricht UMC+, 6200 MD Maastricht, the Netherlands; e Department of Youth Health Care, Living Lab Public Health Mosa, South Limburg Public Health Service, Heerlen, the Netherlands; f Department of Medical Microbiology, Infectious Diseases and Infection Prevention, Care and Public Health Research Institute (CAPHRI), Maastricht University Medical Centre (MUMC+), 6202 AZ Maastricht, the Netherlands

**Keywords:** Migrants, Young people, Access to sexual health care, Consultation rates

## Abstract

**Introduction:**

Migration is a growing phenomenon and has impact on sexual and reproductive health outcomes, such as an increased burden for STIs, sexual violence and unintended pregnancies. Equitable access to sexual health care is of great importance for young people from ethnic minorities (EMs). In this study, we aimed to determine the proportional representation of first- and second generation EMs under 25 years at Dutch Sexual Health Centers (SHCs) compared to native Dutch citizens.

**Methods:**

In this retrospective cohort study, coded health records data of 270,927 persons in the age group of 15 till 24 years visiting SHCs between 2016 and 2021 were included. Health records data was combined with census tract data (Statistics Netherlands) to average annual calculate consultation rates, i.e., dividing 6-year-average of the number of first consultations of a patient in the study period belonging to a specific EM by the total number of citizens in the age group of 15 till 24 years belonging to that EM in the Netherlands in 2021, multiplied by 1000.

**Results:**

The consultation rate for native Dutch patients was 22.0 per 1000 persons (95 %CI: 21.8–22.2, 18.9, 19.8 (95 %CI: 19.8–20.4) for first-generation EMs and 18.4 (95 %CI: 18.0–18.8) for second-generation EMs. In both first- and second generation EMs, consultation rates for patients from Turkey, Morocco, Eastern Europe and Asia were lower than for native Dutch patients. Consultation rates among patients from Africa were lower for first-generation EMs than native Dutch patients. Consultation rates among patients from Indonesia, Suriname/Dutch Antilles, Latin America and other western countries were equal or higher than among native Dutch patients

**Discussion:**

Our study showed that several EMs were underserved in Dutch sexual health care, suggesting lower access to care and indicating the need for culturally sensitive approaches to increase access. Using consultation rates is informative to indicate inequalities in access to sexual health care among EMs.

## Highlights

• In both first- and second generation ethnic minorities, consultation rates for patients from Turkey, Morocco, Eastern Europe and Asia were lower than for native Dutch patients, indicating underrepresentation of several ethnic minorities at Dutch sexual health care.
• Using consultation rates is informative to indicate inequalities in access to sexual health care among ethnic minorities.
• Incidence rates in first repeat visits were higher among first- and second-generation ethnic minorities, suggesting that ethnic minorities might not be disadvantaged in continued use of sexual health care.

## Introduction

During the last decades, migration is a growing phenomenon. Migrant stock data showed that nearly 87 million international migrants lived in Europe, an increase of nearly 16 percent since 2015 (IOM. World Migration Report 2024). Migration can have impact on sexual health and reproductive outcomes in young people, leading to a higher burden of sexually transmitted infections (STIs), unintended pregnancies and sexual violence (Botfield et al., 2016). This vulnerability arises from diverse cultural, political and psychosocial factors, such as stigma and discrimination, poor travel, living and working conditions, and increased exposure to violence and exploitative conditions (Egli-Gany et al., 2021). Migrants also face unequal access to sexual healthcare and a lack of continuity of care, affecting STI testing and treatment, contraceptive services, and vaccinations, such as those for human papillomavirus (HPV) (Botfield et al., 2016; Davies et al., 2006). European-American studies have showed higher STI burden among several first-generation and second-generation migrant groups. A UK study established a significant association between ethnic origin and reported STIs in the previous five years (Aicken et al., 2020). A USA study assessed that African Americans are nearly five times more likely to be infected with an STI compared to other ethnic groups (Laumann and Youm, 1999). In the Netherlands, several studies have also shown that STI positivity rates were higher among individuals with an ethnic minority (EM) background compared with the native Dutch population, especially among EMs under the age of 25 years (van Oeffelen et al., 2017; Ostendorf et al., 2021; Kayaert et al., 2023). Individuals under the age of 25 years from regions or continents such as Morocco, Turkey, the Dutch Antilles, Suriname, Africa and Eastern Europe are particularly at higher risk of *Chlamydia trachomatis (CT)* and *Neisseria gonorrhoeae (NG)*(9)*.* This disparity in STI risk is partly attributed to the endemic presence of STIs in these countries, transnational sexual networks, and unequal access to healthcare services (Laumann and Youm, 1999; Hughes and Field, 2015).The higher burden of sexual health problems underpins the importance of equal access to sexual health services among EMs.

A national survey study from the UK demonstrated that health services are less used by EMs compared with resident populations (Saunders et al., 2021). A literature review demonstrated that structural challenges associated with health system navigation and knowledge of services, expected and perceived stigma, and insufficient culturally safe and language-specific services posed significant barriers to healthcare (Machado et al., 2021). Underutilization of healthcare services was particularly relevant in cases of specialized care, such as sexual health care. In the Netherlands, sexual health care is organized by general practitioners and Centers for Sexual Health (SHC). SHC offer free services, including STI/HIV testing, contraceptive care, vaccinations, and counselling to high-risk groups (e.g. young people under the age of 25 years). Several Dutch studies reported lower consultation rates at SHC among EMs aged under 25 years from Turkey, North-Africa, Eastern Europe, and Asia compared to the Dutch native population of this age group, indicating a lower proportional representation of several young EMs at sexual health services (van Oeffelen et al., 2017; Ostendorf et al., 2021). Both studies were performed in specific Dutch regions, limiting their generalizability. Equitable access to sexual healthcare are especially relevant to young people due to their relative higher risk on STIs and unintended pregnancies. Culturally sensitive prevention and care activities tailored at EMs could help to increase access and reduce the STI burden and improve sexual and reproductive health (Furegato et al., 2016). To effectively target these initiatives, updated and broader generalizable data on the representation of young EMs in sexual healthcare across diverse urban areas is needed. Therefore, the current study aims to determine the proportional representation using consultation rates for several first generation and second generation EMs aged under 25 years visiting Dutch SHCs in both urban and rural areas and compare these to consultations rates among native Dutch individuals. Additionally, this study aims to gain insight into continued use of sexual health care by assessing incidence rates of repeat visits in EMs compared to native Dutch individuals.

## Methods

### 2.1 Study design

In this retrospective cohort study, coded electronic health records of patients under the age of 25 years were included from all outpatient Public Health Sexual Health Centers (SHCs; also known as public health STI clinics) in the Netherlands (from 25 Public Health Services with 38 STI clinic locations spread across the country). Reporting of coded patient consultation data to the National Institute for Public Health and the Environment is standardized and mandatory for all SHC thereby covering virtually all consultations performed. The publicly funded Dutch SHC serve high-risk groups, including young people under the age of 25 years, men who have sex with men, and sex workers, offering free STI testing and sexual health counselling on for example condom use, use of contraceptives, sexual pleasure, sexuality and sexual identity, unwanted sexual experiences, and vaccinations (e.g. HPV). We extracted person-level data on sociodemographic characteristics (i.e. region of birth of patient and parents, age, gender and urbanity of the visited SHC). We used first-time consultations of patients and first repeat visits, using a patient identifier, in the timeframe of 2016, January 1st, to 2021, December 31th. First-time consultations include the first visit of a patient during the study period using a unique patient number. In addition to SHC data, we used nationwide census tract data from Statistics Netherlands (CBS) to calculate and compare consultation rates (www.cbs.nl).

### 2.2 Outcomes

#### 2.2.1 EM groups and consultation rates

First generation EMs were defined as individuals born outside of the Netherlands. Second generation EMs were defined as individuals born in the Netherlands of whom one or both of their parents were born outside of the Netherlands. If both parents were born in different EM groups, the classification was determined by the mother’s birth country.

The classification of EMs was based on the classification of Statistics Netherlands, and the following EM groups were identified: western (West, North and South Europe, United States, Canada, Virgin Island, Newfoundland, Greenland and Oceania), and non-western regions, including Asian (Asia, excluding Turkey and Indonesia), East European (Bulgaria, former Soviet Union, former Yugoslavia, Hungary, Poland, Romania, Slovakia, Czech Republic), African (Africa, excluding Morocco), Latin American (North, Central and South America, excluding the United States and Canada), Surinamese and Dutch Antillean (Suriname and the Dutch Antilles), Turkish (Turkey), Indonesian (Indonesia) and Moroccan (Morocco).

### 2.3 Data analysis

Descriptive statistics were used to describe the sociodemographic characteristics (age, gender, urbanity) of the study population. To calculate average annual consultation rates (called consultation rates further in text) per 1000 persons for each EM group, we first summed the number of first consultations of a patient belonging to a specific EM in the age group of 15 till 24 years in the 6-year study period. This number was then divided by 6 to obtain an average annual number of consultations. We used a 6-year average rather than data from a single year to ensure more stable and reliable estimates, particularly for migrant groups with low consultation numbers. Subsequently, this average of first consultations of patients belonging to a specific EM was divided by the total number of inhabitants in the age group of 15 till 24 years belonging to that EM in the Netherlands in 2021 (January 1st till December 31th), multiplied by 1000. Only the most recent year for the total number of inhabitants per EM group in the census tract data was chosen, as these numbers remained relatively stable over time during the study period. Age of patients from the SHC data was determined as the date of visit minus the birth date. Used information on inhabitants belonging to an EM group can be found in Appendix I. We calculated 95 % confidence intervals (95 %CI) for these rates using standard methods for single rates (http://vassarstats.net/prop1.html). Consultation rates for EM groups were calculated separately for male and female EMs. General consultation rates were calculated separately for patients living in highly urban and less urbanized areas. As postal codes were largely missing for EMs in SHC data, level of urbanization was determined by the location of the consulted SHC. SHC in the provinces of Noord-Holland, Zuid-Holland and Utrecht were classified as highly urban, considering the highest environmental address density and also known as the urban conglomerate ‘Randstad’. The provinces of Noord-Brabant, Zeeland, Limburg, Gelderland, Overijssel, Friesland, Groningen, Drenthe and Flevoland were classified as less urban, considering the lower environmental address density. Consultations rates were compared in different first- and second generation EM groups and the native Dutch population (as a reference group) using the absence or presence confidence interval overlap.

Furthermore, incidence rates of first repeat visits were calculated by dividing the number of first repeat visits after the initial STI clinic visit during the study period by the total number of person years (exposure time) in EMs, multiplied by 1000. Exposure time was defined as the time between the first consultation until the first repat consultation or the end of the study period. We again calculated 95 %CI for these rates, and rates were compared by the absence or presence of the intervals.

Statistical analyses were performed using IBM SPSS Statistics (Version 27.0, Armonk, NY, USA).

### 2.4 Ethical approval

After official review, the Medical Ethics Committee of Maastricht University Medical Centre (MUMC+ METC 2017–0251) waived the requirement for formal ethical approval by prevailing laws in the Netherlands and confirmed that written informed consent was not needed because the data originated from standard care, were deidentified, and were analysed anonymously.

## Results

The comparative national population of 15 to 24 year olds was 2139,691 in 2021, based on the nationwide census tract data from Statistics Netherlands (CBS) (Appendix I). The proportion of EMs in the population was 28.5 % (609,902), and the proportion of non-western EMs was 22.5 % (481,676). The proportion of native Dutch people was 71.5 % ((IOM. World Migration Report 2024),529,772), 10.5 % (225,411) for first-generation EMs, and 18.0 % (384,508) for second-generation EMs.

The SHC study population consisted of 270,927 first-time consultations among patients under the age of 25 years between 2016 and 2021, after exclusion of 859 consultations (0.3 %) in which information on region of birth was missing. Of the patients included in the study population (*N* = 270,927) two-third were women (63.7 %), 36.2 % were men and 0.1 % were transgender. Median age was 21 years with an IQR of 20 to 22 years. More than half visited a SHC in highly urban areas (55.0 %; 149,054) and 45.0 % (121,873) in less urban areas. The proportion of EMs among SHC patients under the age of 25 years was 25.5 % (69,179) and the proportion of non-western EMs was 18.7 % (50,737). The proportion of native Dutch patients was 74.5 % (201,748), 9.9 % ((Souleymanov et al., 2023),730) for first-generation EMs, and 15.7 % (42,449) for second-generation EMs.

### 3.1 Consultation rates for total EMs

The consultation rate for native Dutch people was 22.0 per 1000 persons (95 %CI: 21.8–22.2). The consultation rate for all EMs, both first- and second-generation, was 18.9 (95 %CI: 18.6–19.2), for first-generation EMs 19.8 (95 %CI: 19.8–20.4) and for second-generation EMs 18.4 (95 %CI: 18.0–18.8). In non-western EMs, the consultation rate was 17.6 (95 %CI: 17.2–18.0), 17.1 (95 %CI: 16.5–17.7) in first-generation EMS and 17.8 (95 %CI: 17.3–18.3) in second-generation EMs. In highly urban areas, the consultation rate for all EMs was 19.0 (95 %CI: 18.6–19.5), 19.0 (95 %CI: 18.5–19.5) for non-western EMs, and 27.7 (95 %CI: 27.3–28.1) for native Dutch people. In less urban areas, the consultation rate for all EMs was 12.6 (95 %CI: 12.2–13.0), 14.8 (95 %CI: 14.2–15.4) for non-western EMs, and 18.1 (95 %CI: 17.8–18.4) for native Dutch people.

### 3.2 Consultation rates for different EM groups

**Fig. 1:**
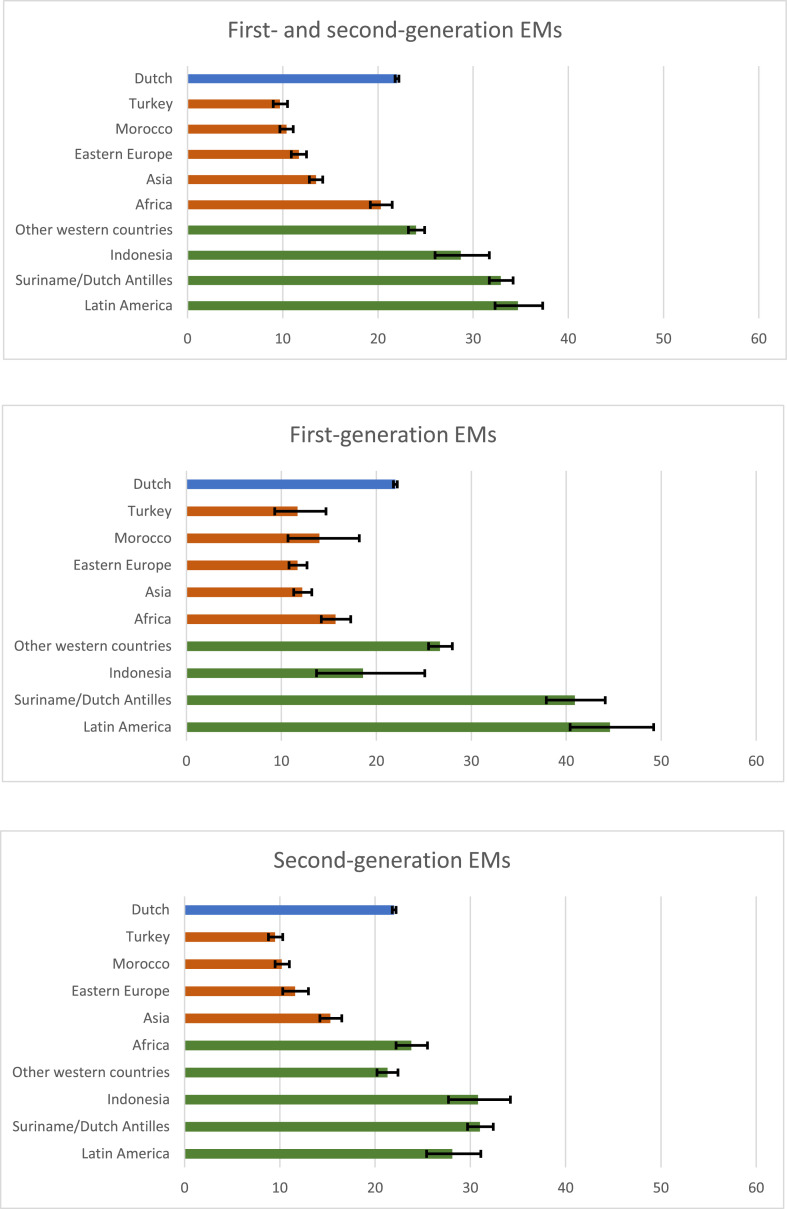
Consultation rates and 95 % confidence intervals compared between native Dutch people and ethnic *minorities* Note: The blue bar represents the reference category (i.e., native Dutch people). A red bar indicates that the consultation rate is significantly lower than that of the reference category (its confidence interval is lower and does not overlap with the confidence interval of the reference group). A green bar indicates that the consultation rate is significantly higher or not significantly different from the reference category (its confidence interval is higher or overlaps with that of the reference group). Frequencies used to calculate consultation rates can be found in Appendix I.

In both first- and second generation EMs, consultation rates for people from Turkey, Morocco, Eastern Europe and Asia were lower than for native Dutch people. Consultation rates among people from Africa were lower for first-generation EMs, but not for second-generation EMs than native Dutch people. Consultation rates among people from Indonesia, Suriname/Dutch Antilles, Latin America and other western countries were equal or higher than among native Dutch people (Fig. 1).

### 3.3 Consultation rates for male and female EM groups

**Fig. 2:**
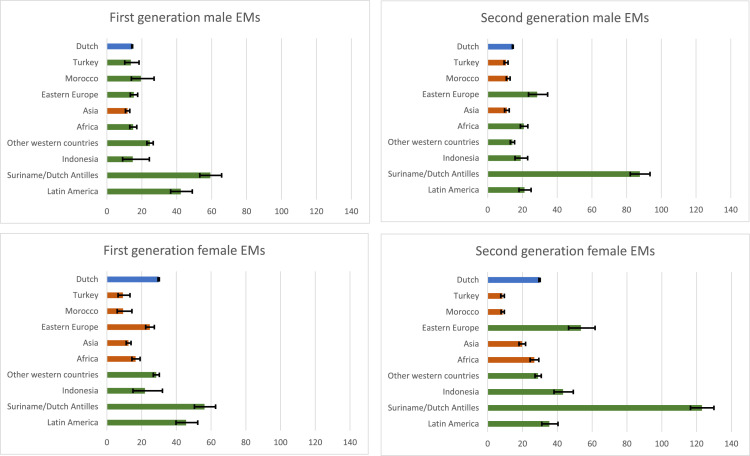
Consultation rates and 95 % confidence intervals compared between native Dutch people and ethnic minorities in men and women Note: The blue bar represents the reference category (i.e., native Dutch people). A red bar indicates that the consultation rate is significantly lower than that of the reference category (its confidence interval is lower and does not overlap with the confidence interval of the reference group). A green bar indicates that the consultation rate is significantly higher or not significantly different from the reference category (its confidence interval is higher or overlaps with that of the reference group). Frequencies used to calculate consultation rates can be found in Appendix II and III.

The consultation rate for native Dutch male patients was 14.5 (95 %CI 14.2–14.7) and for native Dutch female patients 29.8 (95 %CI 29.4–30.2). The consultation rate for all first-generation male EMs was 18.3 (95 %CI 17.5–19.1) and for first-generation female EMs 21.1 (95 %CI 20.3–22.0). For all second-generation EMs, the consultation rate was 17.4 (95 %CI 16.8–18.1) for men and 13.1 (95 %CI 12.9–13.2) for women. The consultation rate for first-generation non-western male EMs was 11.4 (995 %CI 11.1–11.7) and for non-western female EMs 19.8 (95 %CI 18.6–21.0). For second-generation non-western EMs, the consultation rate was 23.9 (95 %CI 23.0–24.9) for men and 19.9 (95 %CI 19.1–20.6) for women. For women, consultation rates among patients from Turkey, Morocco, Asia and Africa or who had a parent from these areas were lower than native Dutch women. The consultation rate among men from Asia or with a parent from Asia was lower than among native Dutch people (Fig. 2).

### 3.4 Incidence rates of first repeat visits for total EMs

The incidence rate of first repeat visits for native Dutch people was 116 (95 %CI: 115 – 117) per 1000 person years. The incidence rate was 124 (95 %CI: 121–127) for all first-generation EMs and 146 (95 %CI: 144–149) for second-generation EMs. Among non-western EMs, the incidence rate was 139 (95 %CI: 135–142) in first-generation EMs, and 153 (95 %CI: 150–155) in second-generation EMs.

## Discussion

This study shows that consultation rates at SHC were generally lower among non-western EMs than native Dutch patients under the age of 25 years, in both first- and second-generation EMs. Consultation rates were especially lower among patients with an Asian, Eastern European, Moroccan, and among Turkish first- or second-generation migration background. For first-generation EMs, the consultation rate was also lower among patients with an African migration background. Although consultation rates in highly urban areas were generally higher than in less urban areas for both native Dutch patients and EMs, consultations rates among EMs were lower than in native Dutch people in both urban and less urban areas. Consultation rates among men were generally lower than among women, but lower consultation rates among non-western EMs were assessed for both men and women. Lower consultations rates from patients with specific EM backgrounds were more visible in women. Repeat visits were higher among (non-western) EMs compared to native Dutch patients, suggesting that EMs might not be disadvantaged in continued use of sexual health care. This suggests that first access to sexual health care might be suboptimal for young EMs and indicates the importance of culturally sensitive approach to sexual health promotion and service provision in both high and less urban areas.

Previous studies have already indicated disparities in access to sexual healthcare among migrants and EM. The current study results are comparable to previous Dutch studies in SHCs in specific highly urban areas and in a less urban area showing lower consultation rates for patients with an Asian, Eastern European, Moroccan, Turkish and African ethnic background and higher consultation rates for patients from Latin America and Suriname/Netherlands Antilles (van Oeffelen et al., 2017; Ostendorf et al., 2021). However, in these previous studies, no distinction was made between first- and second generation EMs. Although our study showed some differences in the proportional representation of specific EMs at SHCs, the pattern of EMS with lower and higher consultation rates was comparable for first- and second generation EMs, in both men and women. This suggests that barriers in accessing sexual health care are still present in persons born in the Netherlands with parent(s) born in the above listed countries. A previous Dutch study in general practitioners (GP) data showed higher consultation rates for EM, except for Turkish and Moroccan women, suggesting that some EM prefer consulting the GP over SHCs (Woestenberg et al., 2015). Our study shows that consultation rates were generally lower among men than among women, which was in line with national surveillance reports (Kayaert et al., 2023). The lower consultation rates for patients with an Asian, Eastern European, Moroccan, Turkish and African ethnic background were more visible among women than among men. However, as men generally already tend to access the SHCs less and the consultation rates were also low for patients with the above-mentioned ethnic backgrounds, attention for improving access to SHCs should be provided to both men and women.

A scoping review (Adrian Parra et al., 2024) has shown that social and structural determinants play a main role in explaining inequalities in sexual health outcomes and use of sexual health services among EMs. On the political level, economic crises can lead to framing migrants as a threat to the economy and can lead to fear among EMs as ‘using too many resources’ (Bloemraad et al., 2019). During economic crises in Europe, there is evidence of a higher STI risk among EMs (Kentikelenis et al., 2015). In addition, unclear and contradicting policies on requirements such as proof of residence and insurance to access health care, create barriers for both EMs and healthcare providers (Adrian Parra et al., 2024; Jones et al., 2021; Martinez and Ormel, 2024; Bil et al., 2019; Nöstlinger et al., 2022; Pérez-Sánchez et al., 2024). On the socioeconomic level, discrimination, poverty and poor travel, living and working conditions for some EMs could lead to poor sexual health outcomes, such as sexual violence (Adrian Parra et al., 2024; Alessi et al., 2021). Furthermore, there are cultural, religious and linguistic barriers related to healthcare provision and access (Nöstlinger et al., 2022; Barrio-Ruiz et al., 2024; Dillon et al., 2024; Souleymanov et al., 2023). On the individual level, knowledge and attitude towards sexual health services and experienced or expected stigma regarding migrant status and sexual preferences, such as homosexuality, play a role in explaining disparities (Adrian Parra et al., 2024; Martinez and Ormel, 2024; Nöstlinger et al., 2022; Pérez-Sánchez et al., 2024). Furthermore, experiences and familiarity with healthcare systems in departure countries might also shape migrants’ practices in sexual healthcare seeking in transition and destination countries (Kamenshchikova et al., 2024). Based on previous research, participative and culturally tailored approaches taking into account the specific healthcare needs and the barriers on these different levels (Kamenshchikova et al., 2024; Bouaddi et al., 2023; Inthavong and Pourmarzi, 2024; Candeias et al., 2021; Lozano et al., 2023; Cordel et al., 2022) should probably be used to tackle disparities in access to sexual health care among people with an Asian, Eastern European, Moroccan, Turkish and African ethnic background.

The use of a large dataset including diverse EMs visiting sexual healthcare centers in both highly urban and less urban areas combined with national population numbers provides insight in the representation of vulnerable groups at sexual healthcare centers. However, a general limitation of this study is that we were only able to use SHC data, and no data of other sexual healthcare providers, such as general practitioners (GPs). It might be possible that several EMs more frequently visit GPs for sexual healthcare (Woestenberg et al., 2015), possibly due to a lack of knowledge on SHCs, and might not use sexual healthcare less frequently but in other care settings. Nevertheless, access to the free and anonymous SHCs should be equal to all young people regardless of migration background. Another limitation in this study is that it is impossible in population numbers to differentiate between men who have sex with men (MSM) and men having sex with women. Discrimination and persecution surrounding homosexuality exists in several cultures worldwide, such as in a large number of African countries, Southeast Asia, and Eastern Europe (Poushter and Kent, 2020). This might lead to even lower consultation rates and unequal access to sexual healthcare among MSM from these countries.

In conclusion, our study showed that several EMs were underrepresented at the Dutch sexual healthcare centers, including first- and second-generation EMs with an Asian, Eastern European, Moroccan and Turkish ethnic background. Using consultation rates by combining population numbers and sexual health center patient data helps to inform for which population groups targeted approaches are needed to increase access. More research is needed into the factors related to lower consultation rates to inform culturally tailored approaches.

## Data sharing

The sexual health center data used were explicitly made available this study by the National Institute for Public Health and the Environment. Any data sharing requests should be directed to the National Institute for Public Health and the Environment (soap@rivm.nl). The nationwide census tract data are publicly available from Statistics Netherlands (CBS) (www.cbs.nl).

## Funding

This work was supported by the Public Health Service South Limburg, the Netherlands.

## CRediT authorship contribution statement

**Y.J. Evers:** Writing – review & editing, Writing – original draft, Visualization, Supervision, Methodology, Formal analysis, Data curation, Conceptualization. **A. Verhaegh:** Writing – review & editing, Writing – original draft, Visualization, Methodology, Conceptualization. **A. Ibrahim:** Writing – review & editing, Writing – original draft, Methodology, Formal analysis, Data curation, Conceptualization. **C. Peters:** Writing – review & editing, Conceptualization. **N.H.T.M. Dukers-Muijrers:** Writing – review & editing, Supervision. **R. Reijs:** Writing – review & editing, Supervision, Conceptualization. **C.J.P.A. Hoebe:** Writing – review & editing, Writing – original draft, Supervision, Conceptualization.

## Declaration of competing interest

The authors declare no conflicts of interest.

## Figures and Tables

**Table:** 

	**Sexual Health Centers electronic health records data** *Total N=270,927* Number of first visits in a 6-year period from 2016–2021	**Census tract data from Statistics Netherlands^** *Total N=2139,691* Number of inhabitants in 2021	Consultation rates (95 %CI)*
**Dutch**	201,748	1529,772	22.0 (21.8 – 22.2)
**First generation EMs**			
Turkey	432	6179	11.7 (9.3 – 14.7)
Morocco	326	3846	14.0 (10.7 – 18.2)
Eastern Europe	3398	48,259	11.7 (10.8 – 12.7)
Asia	3900	53,482	12.2 (11.3 – 13.2)
Africa	2367	25,117	15.7 (14.2 – 17.3)
Other western countries	10,025	62,500	26.7 (25.5 – 28.0)
Indonesia	245	2199	18.6 (13.7 – 25.1)
Suriname/Dutch Antilles	3797	15,468	40.9 (37.9 – 44.1)
Latin America	2240	8361	44.6 (40.4 – 49.2)
**Second generation EMs**			
Turkey	3546	62,033	9.5 (8.8 – 10.3)
Morocco	4249	69,536	10.2 (9.5 – 11.0)
Eastern Europe	1700	24,394	11.6 (10.3 – 13.0)
Asia	3936	42,944	15.3 (14.2 – 16.5)
Africa	4749	33,298	23.8 (22.2 – 25.5)
Other western countries	8417	65,743	21.3 (20.2 – 22.4)
Indonesia	1972	10,698	30.8 (27.7 – 34.2)
Suriname/Dutch Antilles	11,756	63,263	31.0 (29.7 – 32.4)
Latin America	2124	12,599	28.1 (25.4 – 31.1)

^https://opendata.cbs.nl/statline/#/CBS/nl/dataset/37325/table?ts=1678195852121.

*To calculate the consultation rates, the number of first visits are first divided by 6 to obtain an average annual number of consultations.

**Table:** 

	**Sexual Health Centers electronic health records data** Number of first visits in a 6-year period from 2016–2021	**Census tract data from Statistics Netherlands^** Number of inhabitants in 2021	Consultation rates (95 %CI)*
**Dutch**	67,960	782,520	14.5 (14.2 - 14.7)
**First generation EMs**			
Turkey	258	3143	13.7 (10.2 – 18.4)
Morocco	212	1798	19.5 (14.0 – 27.0)
Eastern Europe	1221	13,265	15.4 (13.4 – 17.6)
Asia	2039	28,816	11.8 (10.6 – 13.1)
Africa	1232	13,695	15.0 (13.1 – 17.1)
Other western countries	4172	28,253	24.6 (22.9 – 26.5)
Indonesia	90	1014	14.8 (9.0 – 24.3)
Suriname/Dutch Antilles	1931	5443	59.2 (53.2 – 65.7)
Latin America	1029	4067	42.3 (36.5 – 48.9)
**Second generation EMs**			
Turkey	2010	31,958	10.5 (9.4 – 11.7)
Morocco	2489	35,394	11.7 (10.7 – 12.9)
Eastern Europe	605	3545	28.5 (23.5 – 34.5)
Asia	1456	22,149	11.0 (9.7 – 12.4)
Africa	2130	17,041	20.8 (18.8 – 23.1)
Other western countries	2872	33,823	14.2 (13.0 – 15.5)
Indonesia	630	5525	19.0 (15.7 – 23.0)
Suriname/Dutch Antilles	4954	9430	87.6 (82.1 – 93.5)
Latin America	825	6502	21.2 (18.0 – 25.0)

^ https://opendata.cbs.nl/statline/#/CBS/nl/dataset/37325/table?ts=1678195852121.

*To calculate the consultation rates, the number of first visits are first divided by 6 to obtain an average annual number of consultations.

**Table:** 

	**Sexual Health Centers electronic health records data** Number of first visits in a 6-year period from 2016–2021	**Census tract data from Statistics Netherlands^** Number of inhabitants in 2021	Consultation rates (95 %CI)*
**Dutch**	133,608	747,252	29.8 (29.4 – 30.2)
**First generation EMs**			
Turkey	168	3036	9.2 (6.4 – 13.3)
Morocco	113	2048	9.3 (5.9 – 14.4)
Eastern Europe	2167	14,618	24.7 (22.3 – 27.3)
Asia	1844	24,666	12.4 (11.1 – 13.9)
Africa	1131	11,422	16.5 (14.4 – 19.1)
Other western countries	5820	34,146	28.4 (26.7 – 30.2)
Indonesia	154	1185	21.9 (15.0 – 32.0)
Suriname/Dutch Antilles	1853	5500	56.2 (50.4 – 62.6)
Latin America	1178	4294	45.6 (39.8 – 52.3)
**Second generation EMs**			
Turkey	1532	30,075	8.5 (7.5 – 9.6)
Morocco	1757	34,142	8.6 (7.7 – 9.6)
Eastern Europe	1092	3395	53.6 (46.5 – 61.7)
Asia	2474	20,795	19.8 (18.0 – 21.8)
Africa	2616	16,257	26.8 (24.4 – 29.4)
Other western countries	5534	31,859	28.9 (27.2 – 30.8)
Indonesia	1341	5173	43.3 (38.1 – 49.2)
Suriname/Dutch Antilles	6780	9180	123.1 (116.5 – 130)
Latin America	1297	6097	35.4 (31.1 – 40.4)

^https://opendata.cbs.nl/statline/#/CBS/nl/dataset/37325/table?ts=1678195852121.

*To calculate the consultation rates, the number of first visits are first divided by 6 to obtain an average annual number of consultations.
